# WhichTF is functionally important in your open chromatin data?

**DOI:** 10.1371/journal.pcbi.1010378

**Published:** 2022-08-30

**Authors:** Yosuke Tanigawa, Ethan S. Dyer, Gill Bejerano

**Affiliations:** 1 Department of Biomedical Data Science, School of Medicine, Stanford University, Stanford, California, United States of America; 2 Stanford Institute for Theoretical Physics, Stanford University, Stanford, California, United States of America; 3 Department of Physics and Astronomy, Johns Hopkins University, Baltimore, Maryland, United States of America; 4 Department of Developmental Biology, Stanford University, Stanford, California, United States of America; 5 Department of Computer Science, Stanford University, Stanford, California, United States of America; 6 Department of Pediatrics, Stanford University School of Medicine, Stanford University, Stanford, California, United States of America; La Jolla Institute for Allergy and Immunology, UNITED STATES

## Abstract

We present WhichTF, a computational method to identify functionally important transcription factors (TFs) from chromatin accessibility measurements. To rank TFs, WhichTF applies an ontology-guided functional approach to compute novel enrichment by integrating accessibility measurements, high-confidence pre-computed conservation-aware TF binding sites, and putative gene-regulatory models. Comparison with prior sheer abundance-based methods reveals the unique ability of WhichTF to identify context-specific TFs with functional relevance, including NF-κB family members in lymphocytes and GATA factors in cardiac cells. To distinguish the transcriptional regulatory landscape in closely related samples, we apply differential analysis and demonstrate its utility in lymphocyte, mesoderm developmental, and disease cells. We find suggestive, under-characterized TFs, such as RUNX3 in mesoderm development and GLI1 in systemic lupus erythematosus. We also find TFs known for stress response, suggesting routine experimental caveats that warrant careful consideration. WhichTF yields biological insight into known and novel molecular mechanisms of TF-mediated transcriptional regulation in diverse contexts, including human and mouse cell types, cell fate trajectories, and disease-associated cells.

## Author summary

Transcription factors (TFs), a class of DNA binding proteins, regulate tissue- and cell-type-specific expression of genes. Identifying the critical TFs in a given cellular context leads to investigating molecular regulatory mechanisms in development, differentiation, and disease. Because there are more than 1,500 human TFs, experimental measurements of genome-wide occupancy across all TFs have been challenging. While computational approaches play pivotal roles, most existing methods rely on statistical enrichment, focusing either on sequence motif similarity recognized by TFs or the similarity of the genomic region of interest with the previously characterized TF occupancy profile. Here we propose WhichTF as an alternative, incorporating curated biomedical knowledge from ontology and integrating it with the high-confidence prediction of conserved TF binding sites in user-provided genomic regions of interest. We develop a new WhichTF score to rank TFs and demonstrate its applicability across human and mouse cell types, cellular differentiation trajectories, and disease-associated cells.

## Introduction

Transcription factors (TFs) are the master regulators of development. They define, refine, and can even divert cellular trajectories. TFs perform these important tasks by binding to specific DNA sequences in open chromatin, where they recruit additional co-factors and together modulate the expression of downstream genes. TFs regulate biological processes in healthy adult tissues, and mutations in both TF genes and their genomic binding sites have been linked with human disease [1,2].

The advent of next-generation sequencing has paved the way for chromatin immunoprecipitation followed by sequencing (ChIP-seq)-based methods for discovering genome-wide loci where a given TF binds DNA in a given cell population [3]. Tools developed for the analysis of ChIP-seq data, such as GREAT [4] (Gene Regulatory Enrichment of Annotations Tool), have discovered and leveraged a compelling phenomenon: when a TF is functionally important for the progression of a certain process, such that its perturbation leads to the disruption of this process, the binding sites for this TF are often highly enriched in the gene regulatory domains of multiple *downstream* target genes that drive this process [4].

TFs work in different combinations to enact a vast repertoire of cellular fates and responses [5]. Between 1,500–2,000 TFs are thought to be encoded in the human genome [1]. Performing ChIP-seq for more than a handful of TFs in any cellular context is an expensive and laborious procedure, while the assaying of hundreds of TFs even in the same cell state is impractical except in a handful of settings mostly in lavishly funded consortia.

To obtain a more comprehensive view of transcriptional regulation in action, experimental focus has turned from the assaying of individual TFs to the assaying of all open chromatin in a given cellular context. These DNase-seq, ATAC-seq, or single-cell ATAC-seq accessibility profiles offer a proxy for all cis-regulatory elements active in a given cellular state [6–8].

While assaying all TFs is infeasible, many hundreds of TFs have been studied in one or more cellular contexts, or via complementary methods (such as protein binding microarrays or high-throughput SELEX), to obtain the DNA binding preference of the TF [1]. Collectively, virtually all TF families, if not all TFs, have their binding profile characterized in some cellular context [1] (S1 Fig). These hundreds of TF binding motifs can then be used to predict transcription factor binding sites (TFBSs) for all characterized TFs in various context-specific sets of accessible chromatin regions.

Very often, biological processes of interest are conserved at the genome sequence level across closely related species, such as primates or mammals. As such, computational tools like PRISM [9] (Predicting Regulatory Information for Single Motifs) can be used to obtain a rarefied subset of binding site predictions that are both observed to be positioned in open chromatin and conserved orthologously in additional species. Because these sites evolve under purifying selection, they are more likely to be individually important in the probed context [9].

Here, we innovate on the foundation of two tools our group previously developed: PRISM [9] to predict conserved binding sites for hundreds of human and mouse TFs, and GREAT [4] to detect enriched functions in gene regulatory regions. We use insights from both approaches to develop WhichTF, a tool that applies a novel statistical test to identify context-specific TFs of functional importance within a set of user-specified open chromatin regions. In this work, dominant TFs refer to TFs whose conserved binding sites are enriched within functionally coherent regions of the input open chromatin regions. In contrast to many existing tools that evaluate the relative abundance of TFBSs against previously characterized epigenomic data tracks or genome-wide backgrounds [10–19], WhichTF leverages curated knowledge about the functional impact of genes and their putative regulatory domains and applies stratified enrichment analysis in genomic regions with functionally coherent annotations to assesses the functional impact of TFs. We compare our ontology-guided functional approach against abundance-based approaches, including existing methods and our PRISM-predicted conserved TFBS enrichment [9]. We show that our molecular definition of dominance successfully predicts biologically important and cell-type-specific factors in the context of different cell types, differentiation pathways, and even disease-associated cellular sets, while enrichment-only based methods tend to highlight TFs with broad functionality across many cellular contexts.

## Results

### Motivation: Sheer abundance-based tools’ performance

To predict dominant TFs, we first tested existing tools using DNase-seq profiles from well-studied human B-cells, T-cells, heart, and brain cells as inputs. Specifically, we applied five prior TF ranking tools, MEME-ChIP from the MEME suite [10], a motif enrichment tool implemented in the regulatory genomics toolbox (RGT) [11], HOMER [12], cisTarget [13], and Genomic Locus Overlap Analysis (LOLA) [14]. Those tools rank TFs based on the enrichment of sheer motif abundance in the input accessible regions or enrichment against previously characterized ChIP-seq measurements (Table 1 and S1 Table, Methods). While those tools identify several of the known TFs, we found their results were often not cell-type specific and dominated by structural TFs (Table 1). For example, MEME-ChIP [10] ranked NFIA and NEUROD2 (also known as NDF2) as the top two hits in the brain, both of which play roles in central nervous system development [20,21]. TWIST1, the 4th ranked TF in both T-cells and B-cells by MEME-ChIP, regulates the proinflammatory response of T helper 1 cells while its function in B-cells is unknown [22]. For the other top-ranked TFs, however, to our best knowledge, there is limited literature support for their cell-type-specific role. Indeed, a known structural TF, CCCTC-Binding Factor (CTCF), and transcriptional repressors, ZIC2 and HIC1, were all identified among the top 5 TFs by MEME-ChIP for at least 3 out of 4 cell types.

**Table 1:** WhichTF identifies cell-type-specific functionally important TFs in diverse cell types.

**a**. B cells (ENCFF719GOE)								
	MEME-ChIP	RGT	PRISM enrichment		**WhichTF**			
			TF	-log_10_(P)	**TF**	**-log**_**10**_ **(CP)**	**Importance**	**PMID**
1	CTCF	ZNF148	CTCF	6414.1	**SPIB**	**76.0**	**Confirmed**	**21057087**
2	ZIC2	KLF5	SP4	5035.1	**NFKB1**	**89.5**	**Confirmed**	**20452952**
3	ZIC1	KLF4	KLF14	4280.1	**RELB**	**62.1**	**Confirmed**	**20452952**
4	TWIST1	KLF15	NFYA	4174.8	**RELA**	**32.0**	**Confirmed**	**20452952**
5	HIC1	SP9	NFYB	4030.4	**SPIC**	**11.5**	**Confirmed**	**21057087**
**b**. T cells (ENCFF861OSQ)								
	MEME-ChIP	RGT	PRISM enrichment		**WhichTF**			
			TF	-log_10_(P)	**TF**	**-log**_**10**_ **(CP)**	**Importance**	**PMID**
1	CTCF	ZNF148	CTCF	7491.4	**NFKB1**	**96.8**	**Confirmed**	**20452952**
2	ZIC2	KLF5	SP4	5784.8	**RUNX3**	**89.1**	**Confirmed**	**12796513**
3	ZIC4	KLF4	KLF14	5101.4	**RELB**	**63.5**	**Confirmed**	**20452952**
4	TWIST1	CTCFL	E2F2	4790.1	**RELA**	**43.0**	**Confirmed**	**20452952**
5	HIC1	KLF15	E2F3	4631.6	**REL**	**15.6**	**Confirmed**	**20452952**
**c**. Heart (ENCFF176HSL)								
	MEME-ChIP	RGT	PRISM enrichment		**WhichTF**			
			TF	-log_10_(P)	**TF**	**-log**_**10**_ **(CP)**	**Importance**	**PMID**
1	NFIX	EWSR1-FLI1	CTCF	6366.2	**GATA5**	**50.5**	**Confirmed**	**16987437**
2	CTCF	ETV4	E2F3	5167.1	**GATA4**	**19.5**	**Confirmed**	**16987437**
3	NEUROD1	CTCF	E2F2	5081.6	**GATA6**	**18.3**	**Confirmed**	**16987437**
4	HIC1	KLF4	ZBTB14	5029.8	**TEAD4**	**10.8**	**Confirmed**	**28178271**
5	PKNOX1	ZNF148	CNOT3	4987.2	**FOS**	**12.1**	**Confirmed**	**16934006**
**d**. Brain (ENCFF318HIS)								
	MEME-ChIP	RGT	PRISM enrichment		**WhichTF**			
			TF	-log_10_(P)	**TF**	**-log**_**10**_ **(CP)**	**Importance**	**PMID**
1	NFIA	EWSR1-FLI1	CTCF	5863.6	**SOX2**	**69.4**	**Confirmed**	**28733588**
2	NEUROD2	ETV4	ZBTB14	5361.9	**OTX1**	**12.5**	**Confirmed**	**20354145**
3	CTCF	ZNF148	E2F3	5341.2	**GLI1**	**16.8**	**Confirmed**	**14581620**
4	ZIC2	ZNF263	E2F2	5258.0	**GLI2**	**8.0**	**Confirmed**	**14581620**
5	HIC1	CTCF	CNOT3	5051.6	**ISL1**	**6.7**	**Confirmed**	**24763339**

The top 5 identified TFs for B-, T-, heart, and brain cells are shown for 4 methods: MEME-ChIP, regulatory genomics toolbox (RGT) motif enrichment tool, PRISM conserved binding site enrichment, and WhichTF. MEME-ChIP, RGT, and PRISM pure abundance-based tools all ranked structural elements like CTCF and other general housekeeping factors high across multiple cell types. In contrast, because WhichTF incorporates context-dependent functional filtering, its top-ranked TFs are all important in their specific functional contexts, as evident by the PubMed IDs we quote. Here, -log_10_(P) denotes the statistical significance (negative log_10_ p-value) of the TFBS enrichment; -log_10_(CP) denotes the statistical significance (conditional p-value, conditioned on the TFs ranked above, Methods); and PMID represents the PubMed ID. Comparisons with additional methods are shown in S1 Table.

We found a similar trend in the results from other abundance-based tools (S1 Table). For RGT motif enrichment [11], Kruppel family factors, KLF4 and ZNF148, were ranked among the top 5 TFs across at least 3 out of 4 cell types (Table 1 and S1 Table). HOMER [12] performs two sets of analyses: enrichment for the known motifs and de novo motif discovery followed by comparison against a reference library of known motifs. For all of the eight conditions across both analysis modes and four cell types, HOMER ranked structural element, CTCF or CTCF-like (CTCFL), as the top-ranked TF (S1 Table). Some of the TFs ranked among the top five have literature-supported cell-type-specific roles. SPIB and SPI1 are in the NF-κB pathway and play roles in lymphocyte development and adaptive immunity [23]. ATOH1 regulates respiratory circuitry development in hindbrain neurons [24] and is ranked next to CTCF (or CTCFL) in the brain by HOMER [12]. LOLA [14] identified histone lysine demethylase, PHF8, for three out of four cell types, as well as other factors involved in chromatin looping, including mediator complex subunit 1 (MED1), RAD21, and CTCF (S1 Table). BRD4, the fifth-ranked TF in T-cells by LOLA [14], regulates the differentiation of pathogen-specific CD8 T cells [25]. The results of cisTarget [13] include RNA polymerase II-related factors, including NRF1 and POLR2A (S1 Table). Overall, those results indicate that the enrichment tests based on the sheer abundance of the sequence motifs in the given genomic regions tend to yield less cell-type-specific results.

We next examined whether incorporating conservation-aware predictions of TFBSs yielded cell-type-specific TFs of functional relevance. To this end, we applied PRISM [9] to predict mammalian conserved TFBSs using 672 manually curated PWMs from 567 TFs, including the members of all major TF families (S1 Fig, S1 Data, Methods) across the entire genome. When we assessed the enrichment of TFBSs in the specified accessible regions, we found that the top-ranked TFs were still dominated by a similar set of non-specific factors, such as CTCF, E2F2, and E2F3, across the different cell types (Table 1, Methods). Motivated by these results, we moved to investigate whether a stratified enrichment that focuses on the functionally important genomic regions would highlight TFs with cell-type-specific functions.

### WhichTF approach overview

**Fig 1:**
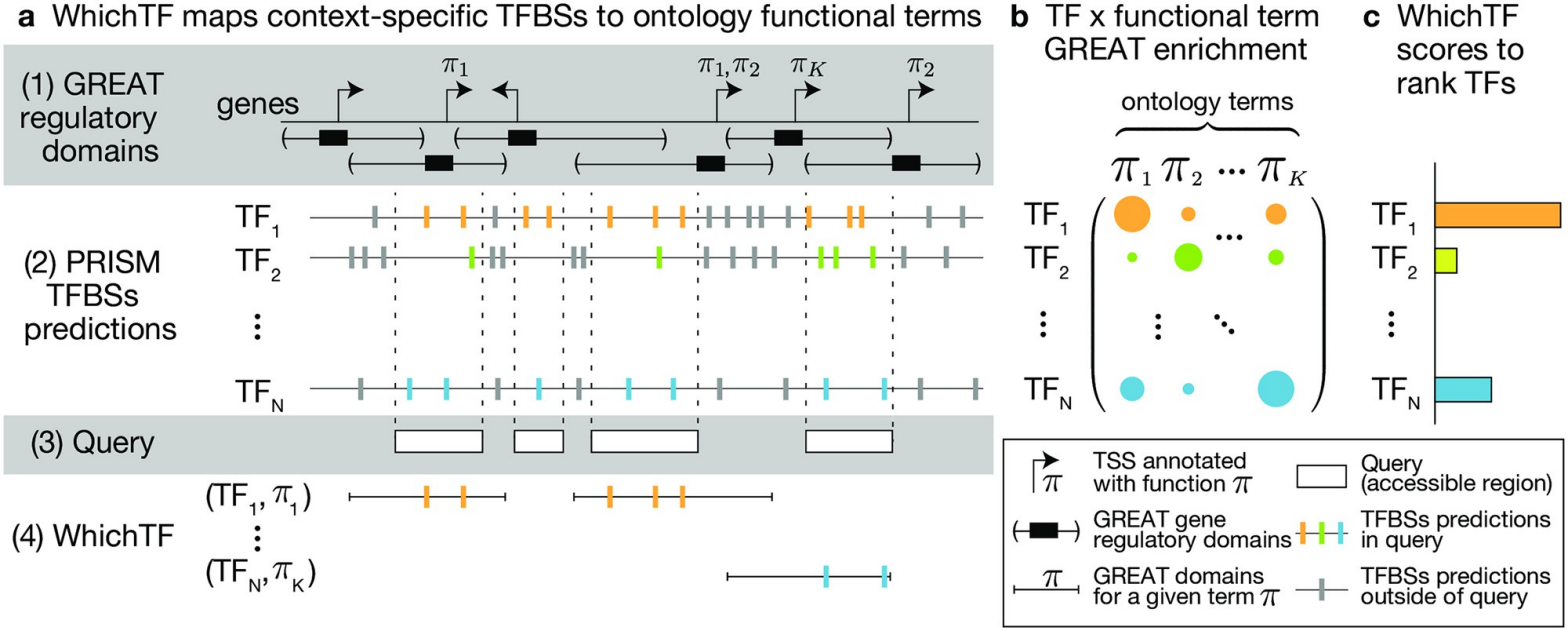
The WhichTF computational model. (a) WhichTF uses gene regulatory domain models and functional information in ontologies from the genomic region enrichment analysis tool (GREAT) (step 1) and conservation-based PRISM predictions of transcription factor binding sites (TFBS) (step 2). Given a user-defined set of putative gene regulatory genomic regions (step 3), WhichTF considers the top-*K* GREAT functional terms (*π*_1_, …, *π*_*K*_) enriched in the query regions. For all pairwise combinations of a top-*K* term and a TF, WhichTF counts the number of TFBSs within the specified query regions (step 4). (b) The binomial and hypergeometric TFBS enrichment *p*-values for each ontology term are compiled in a TF-by-term summary statistic matrix (Methods). (c) Aggregating the summary statistics over terms, WhichTF returns a ranked list of TFs, ordered by predicted functional importance in the user-specific chromatin environment, with the corresponding scores and statistics. WhichTF also reports a set of ontology terms driving each enrichment (Methods). Here, TSS stands for transcription start site.

To provide context-specific predictions, WhichTF integrates functional genome annotations from GREAT [4] on top of the conservation-aware TFBS predictions from PRISM [9] (Fig 1). We use GREAT in conjunction with the mouse genome informatics (MGI) mammalian phenotype ontology [26] to annotate all genes in the human GRCh38 (hg38) and mouse GRCm38 (mm10) genomes with a canonical transcription start site (TSS), a putative gene regulatory domain, and any MGI phenotypes known to be affected by mutations to the associated gene. This procedure yields over 700,000 gene-phenotype relationships for each species (Fig 1a, step 1). The updated PRISM [9] predictions provide 268 million and 161 million putative TFBSs for the human and mouse genomes, respectively (Fig 1a, step 2).

For a given query set of experimentally characterized accessible regions (Fig 1a, step 3), WhichTF integrates these functional annotations to identify functionally dominant TFs. Specifically, WhichTF first applies GREAT [4] to the intersection of a user’s input query with all conserved binding sites from PRISM [9] to identify the top 100 enriched ontology terms. Focusing on the GREAT regulatory domains [4] annotated for the identified functional terms, WhichTF counts for each TF the number of conserved binding sites falling within the intersection of these regulatory domains and the user-specified accessible regions (Fig 1a, step 4). It computes a novel stratified enrichment statistic, the WhichTF partial score (Fig 1b), for each pair of term and TF. Aggregating over the top 100 functional terms, WhichTF ranks TFs, reports the WhichTF score, and evaluates significance based on their functional importance (Fig 1c, Methods). To reduce the biases from parametric model misspecification, we used nonparametric, rank-based statistics to compute the WhichTF score, which is a function of the conserved TFBS enrichment within the putative regulatory regions of the genes annotated for the top 100 enriched terms. We report the statistical significance as a conditional p-value (Methods). Because the WhichTF score is derived from non-parametric statistics, the ranking of the TFs rather than the absolute values of the scores are more informative for the interpretation of the results. The top-ranked TFs identified with this ontology-guided approach are hypothesized to be functionally relevant TFs in a cell exhibiting the measured accessibility profile.

### WhichTF identifies functionally important TFs across diverse cell types

We first assessed the performance of WhichTF using the same four human cell types in Table 1. As opposed to the other methods we examined, we found that WhichTF’s top-ranked TFs are cell-type-specific, and their functions are supported in the literature. In B- and T-cells, we identified TFs in the NF-κB pathway, key factors in lymphocyte development and adaptive immunity [23]. In embryonic heart tissue, we found GATA4, 5, and 6 —known regulators of cardiac development and growth that, when perturbed, have been implicated in human congenital heart disease [27]. In embryonic brain tissue, we found SOX2, a critical regulator of neural progenitor pluripotency and differentiation in embryogenesis and later development, including adult hippocampal neurogenesis [28–30]. These results highlight the benefits of ontology-guided stratified enrichment in identifying TFs with cell-type-specific functions.

To further investigate which functional terms contributed to identifying the TFs and gain insight into the biological basis for the stratified enrichment, we ranked the contributing ontology terms using WhichTF partial scores (Methods). To evaluate the suggested pair of TF and ontology terms, we investigated whether the ontology term appears as an annotation for the TF gene itself. In WhichTF, an ontology term prediction is derived not from the TF gene itself but from the annotations of the putative downstream targets of its predicted conserved binding sites within the input query. Because of this, observing that the TF gene itself is annotated with the same term can be seen as additional support regarding the functional importance of the identified TF in the specific cellular context. Applying this “closed-loop” analysis [9] to the top five ontology terms for the top-ranked TF in each of the four cell types, we find the ontology term annotates the predicted TF in 17 / 20 (85%) cases (S2 Table). For example, we identified “abnormal IgG level” (MGI Mammalian Ontology term, MP:0020174) for ranking SPIB as the top-ranked TF in B cells. Similarly, we found “abnormal T cell differentiation” (MP:0002145) for NFKB1 in T cells, “abnormal heart layer morphology” (MP:0010545) for GATA5 in heart cells, and “abnormal neuron differentiation” (MP:0009937) for SOX2 in brain cells as the top-ranked ontology term for the top-ranked TF in each sample.

To test the utility of WhichTF on mouse datasets and evaluate its performance against an experimental TF importance prediction method, we applied WhichTF to mouse DNase-seq datasets for four cell types and compared our results to the active TF identification (ATI) assay [31] for two overlapping cell types. ATI attempts to identify context-relevant TFs by quantifying the total number of TF-DNA binding events in cells or tissues, identifying motifs present in TF-bound DNA, and identifying specific TFs through mass spectrometry of bound protein. We found the overlap of the identified TFs from the two approaches to be minimal (S3 Table). The ATI assay mainly identified TF families rather than individual family members. In heart cells, ATI identified a single context-specific TF family, Thra, while WhichTF yields multiple TFs, including multiple individual Gata factors with literature support (S3c Table). In brain cells, ATI discovered Egr1 and three additional TF families as context-specific TFs, of which Egr1 is known for regulatory roles in synaptic plasticity and neuronal activity [32]. Both POU and SCRT families have member TFs confirmed to be relevant in the brain. The remaining DBP/HLF family does not have literature support and is also identified as a context-specific TF in liver cells [31]. In contrast, WhichTF identifies Zic2 and Sox2 with literature-confirmed context-specific functions as well as suggestive and novel TFs (S3d Table). Both approaches yield context-specific TFs with independent support in the literature, suggesting both methods can complement each other.

To examine the robustness of our approach, we repeated the analysis with varying parameters, including the number and the length of the input regions and the number of the most enriched ontology terms (Methods). We found that the identified top TFs were stable across a broad range of these parameters (S4a–S4c Table).

Taken together, WhichTF can robustly integrate user-specified accessible regions, pre-computed conserved TFBS libraries, and functional information through ontology annotations to identify functionally relevant TFs as well as specific ontology terms contributing to their ranking. Compared with prior *in silico* and *in vitro* TF ranking methods in diverse contexts across species, WhichTF readily discovers cell-type-specific TFs supported by literature and missed by existing approaches.

### WhichTF quantifies biologically meaningful similarities and differences in TF-mediated transcriptional programs

**Fig 2:**
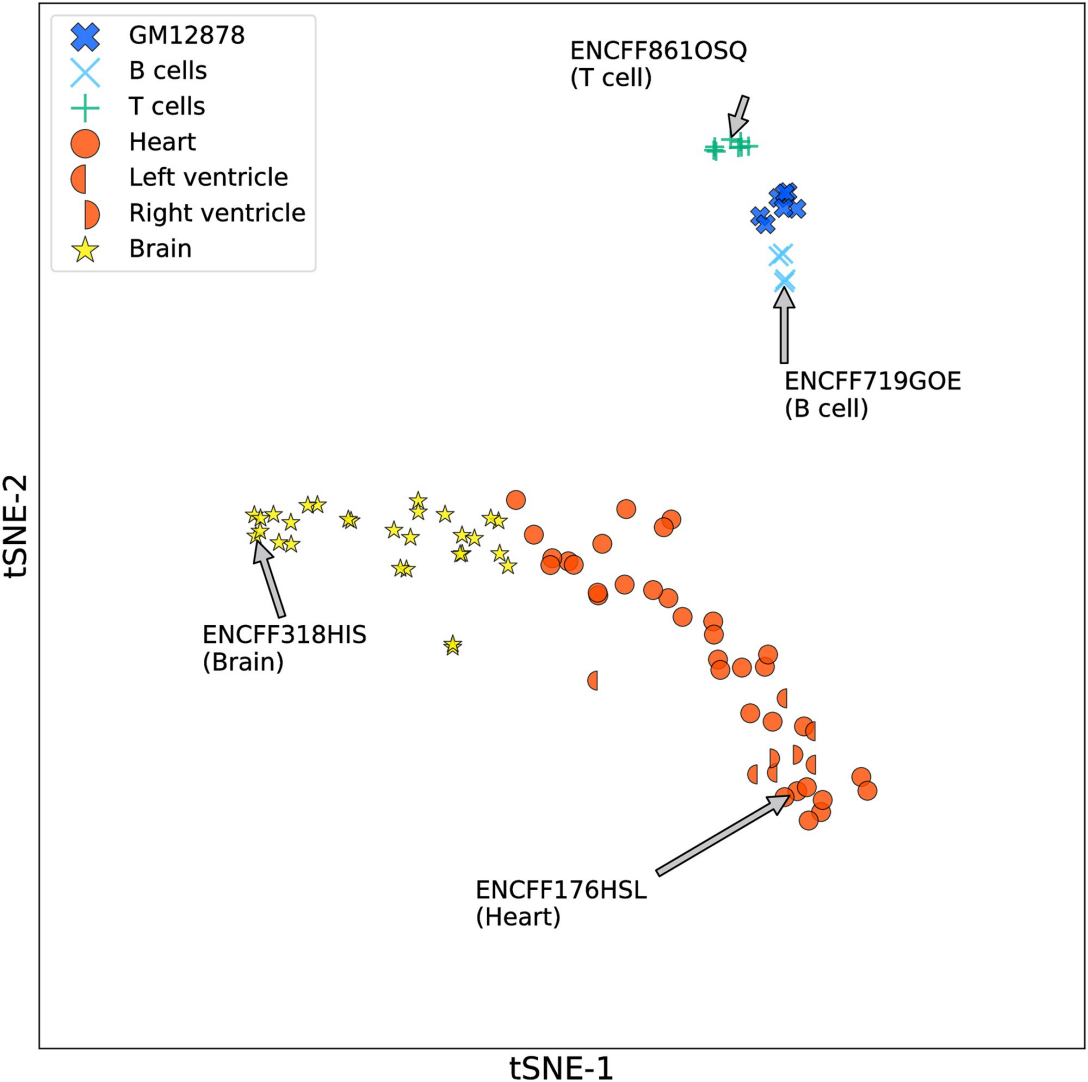
WhichTF captures biological similarities and dissimilarities of TF-mediated transcriptional programs. The WhichTF score vectors are projected to a t-SNE plot for DNase-seq data tracks of 90 samples across 7 cell types, including GM12878 (number of samples, n = 7), B cells (n = 4), T cells (n = 8), Heart (n = 34), Left ventricle (n = 6), Right ventricle (n = 2), and Brain (n = 29). Reassuringly, samples from the same cell types are projected together in the low-dimensional space. The samples analyzed in Table 1 are annotated here with arrows. The raw data used in the figure is available in S2 Data.

Precise knowledge of cell state and identity is crucial for understanding normal development and disease. To assess whether WhichTF can quantitatively capture biologically meaningful similarities and differences in TF-mediated transcriptional programs, we applied a t-distributed stochastic neighbor embedding (t-SNE) [33] to WhichTF score vectors across TFs computed for 90 human samples across seven cell types. We found that brain, lung, and hematopoietic cells are mapped to distinct regions with fine-grained substructures among closely related samples (Fig 2). For example, we observed a clear separation of GM12878 (a lymphoblastoid cell line), B-cells, and T-cells. Reassuringly, different samples from the same biological tissue, such as the left ventricle, the right ventricle, and the heart, clustered together.

### Differential WhichTF identifies differentially dominant TFs in closely related cell types

B-cells and T-cells share a closely related developmental trajectory [23]. As Table 1 shows, prior computational TF ranking tools and PRISM [9] TFBS enrichment did not identify cell-type-specific factors yielding highly overlapping sets of TFs for B-cells and T-cells. In contrast, while WhichTF identified NF-κB family members NFKB1, RELA, and RELB as shared dominant TFs, WhichTF also identified literature supported lineage-specific factors, such as SPI-B for B-cells and RUNX3 for T-cells (Table 1a and 1b). SPI-B is an ETS family TF known to play a key role in B-cell development and function and environmental response [34–36]. RUNX3, in contrast, plays T-cell-specific functional roles, such as in CD4 versus CD8 thymocyte commitment, helper versus killer T-cell specification, and helper type selection [37]. These differential roles for SPI-B and RUNX3 are corroborated by their cell-type-specific expression in B-cells and T-cells, respectively (S2 Fig) [38].

Although we identified multiple TFs distinguishing B- and T-cells, the results still contain many common factors. This is reasonable, as the two cell types share most of their developmental program [23]. To identify TFs with relative dominance in one cell type versus another, we applied differential analysis focusing on uniquely accessible regions only in one type of cell and not the other (Methods). In B-cells (vs. T-cells), the differential analysis highlighted additional ETS family members, PU.1 and SPI-C. These TFs are essential for healthy B-cell differentiation and function (Table 2a). In T-cells (vs. B-cells), we saw an additional RUNX family member, RUNX1 (Table 2b), which is functionally relevant in T-cells. Indeed, RUNX1 and RUNX3 form a complex and are crucial for the healthy function of T-lymphocytes [37,39]. We compared these results with the differential analysis implemented in MEME-ChIP [10] (Methods). In B-cells (vs. T-cells), MEME-ChIP identified two TFs with well-characterized B-cell specific functions, BCL11A and SPI1. The latter of which was also identified by WhichTF. In T-cells (vs. B-cells), however, the results from MEME-ChIP were dominated by TFs involved in general housekeeping processes, such as CTCF, for which we were unable to find clear literature supporting cell-type-specific function in T-cells (Table 2).

**Table 2:** The differential TFs identified from the differential WhichTF analysis applied on B and T-cell DNase-seq data.

**a.** B-cell vs. T-cell						
	WhichTF				MEME-ChIP	
	TF	-log_10_(CP)	Importance	PMID	TF	E-value
1	SPIB	28.3	Confirmed	21057087	BCL11A	8.4 x 10^−62^
2	SPI1	21.3	Confirmed	21057087	SPI1	6.8 x 10^−13^
3	SPIC	17.1	Confirmed	21057087		
4	REL	4.3	Confirmed	20452952		
5	RELB	2.7	Confirmed	20452952		
**b.** T-cell vs. B-cell						
	WhichTF				MEME-ChIP	
	TF	-log_10_(CP)	Importance	PMID	TF	E-value
1	RUNX3	171.1	Confirmed	12796513	CTCF	4.3 x 10^−46^
2	NFKB1	47.7	Confirmed	20452952	MET32	2.5 x 10^−20^
3	RUNX1	36.4	Confirmed	12796513	ZIC2	2.3 x 10^−12^
4	REL	8	Confirmed	20452952	ZIC3	7.3 x 10^−9^
5	CBFB	9.1	Confirmed	17185462	KLF8	1.1 x 10^−5^

The top 5 TFs identified for (a) B-cells relative to T-cells (B-cell minus T-cell) and (b) vice versa (T-cell minus B-cell) are shown with the corresponding statistical significance, negative log_10_ conditional probabilities (-log_10_(CP)). The results from a MEME-ChIP differential analysis are shown for comparison. The E-value is a statistical significance reported from MEME-ChIP and it represents the expected number of motifs in the randomized background set (Methods). The importance and PubMed ID (PMID) columns show that existing literature supports all WhichTF identified TFs, suggesting input data from less well-characterized cell types is likely to produce tangible functional hypotheses.

### WhichTF identifies differentially dominant TFs along developmental trajectories

**Fig 3:**
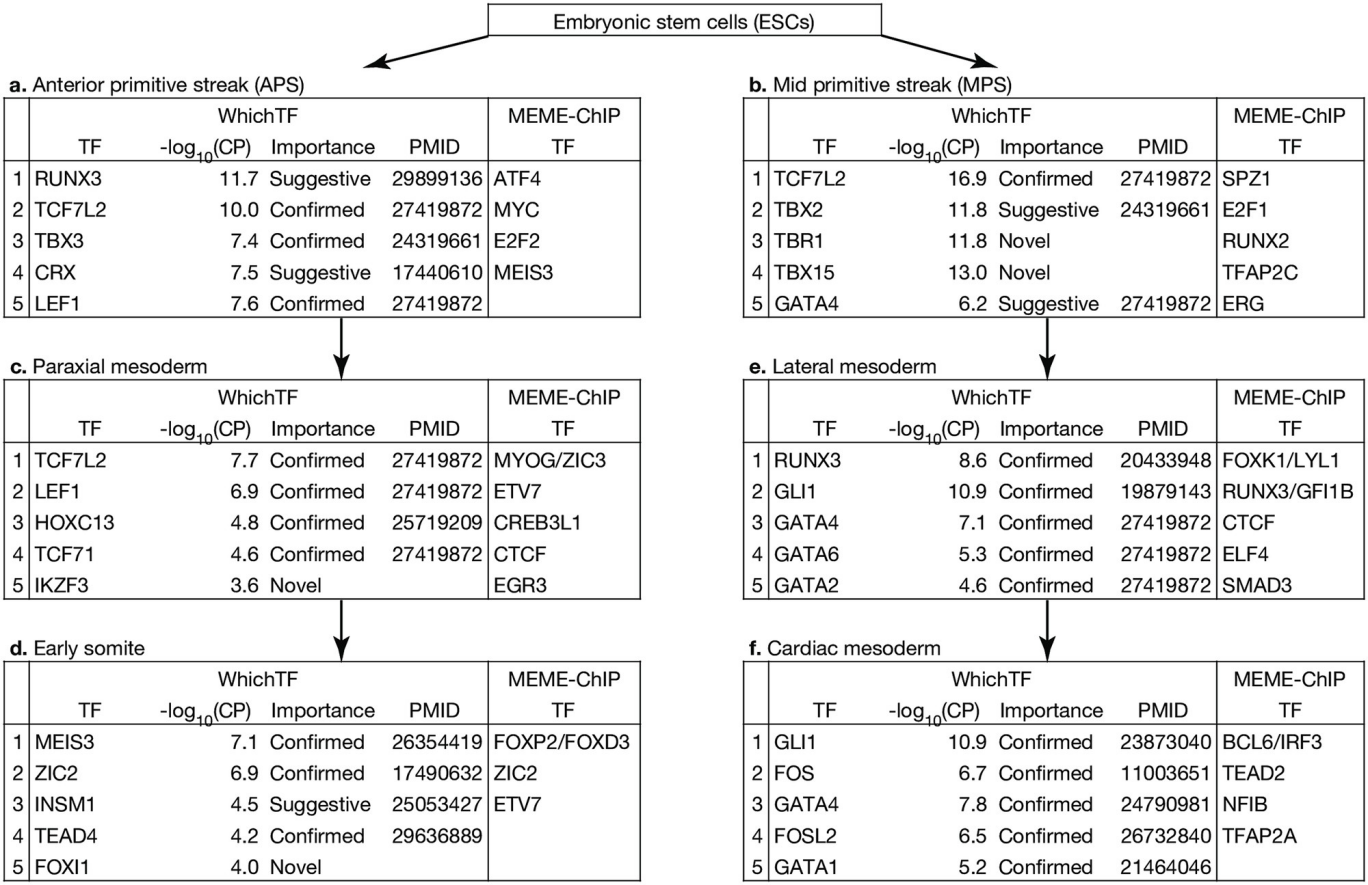
Differential WhichTF analysis identifies differentially dominant TFs compared to immediate progenitor cells along experimentally derived human mesoderm development pathways from ATAC-seq data. The top 5 TFs with their corresponding statistical significance, negative log_10_ conditional probabilities, -log_10_(CP), are shown. The results from a MEME-ChIP differential analysis are shown for comparison. The importance and PubMed ID (PMID) columns indicate whether (i) existing literature supports the identified TFs (confirmed); (ii) literature reports closely related factors, such as co-factors and functionally related family members, or the identified TFs in a related context (suggestive); or (iii) novel. Differential WhichTF and differential MEME-ChIP are found to produce very different predictions. The abundance of confirmed WhichTF predictions (here and in previous figures) makes suggestive and novel predictions more attractive.

TFs regulate cell fate decisions in animal developmental programs [1]. To gain insights into the molecular mechanisms influencing cellular differentiation by identifying differentially dominant TFs, we applied differential analysis to ATAC-seq data collected in experimentally derived mesoderm development trajectories from human embryonic stem cells (ESCs) to early somite and cardiac mesoderm [40] (Fig 3). We applied differential WhichTF and differential MEME-ChIP [10] and compared their results.

The first step of mesoderm development is the differentiation from ESCs to anterior (APS) or mid (MPS) primitive streak (PS) cells. In both APS and MPS cells, we found WNT signaling TFs, such as TCF7L2 and LEF1, and T-box family TFs, such as TBX-2 and -3, were uniquely identified with WhichTF (Fig 3a and 3b). WNT signaling is involved in PS differentiation and is crucial in inducing PS cell types [40]. The set of ontology terms driving the TF ranking is consistent with the cellular contexts we examined. TCF7L2, which is ranked first and second in MPS and APS, respectively, is driven by functionally coherent terms, such as abnormal mesoderm morphology (MP:0014141), abnormal somite development (MP:0001688), and abnormal primitive streak morphology (MP:0002231) (S5a Table) in MPS cells, while being driven by less specific terms, such as abnormal germ layer morphology (MP:0014138) in APS cells (S5b Table). T-box family members also play key roles in PS development. TBX6 is a canonical PS marker, and the specific loss of *Eomes* (a.k.a. *Tbr2*) causes ectopic primitive streak formation in mice [40,41]. The specific T-box family member TBX3, which was ranked third in APS cells, has been implicated in the early stage of differentiation towards mesoderm from ESCs in mice and *Xenopus*. It has been reported for its functional redundancy with Tbx2 during *Xenopus* gastrulation [42]. RUNX3, our top hit for APS, shows conserved expression in mouse neuromesodermal progenitor (NMP) cells and human D3-NMP-like cells. Interestingly, we also found previously unreported T-box family TFs, TBX15 and TBR1, of which TBX15 is linked to decreased skeletal muscle mass in mouse [26] and known for tissue-specific expression in muscle, a tissue developed from the mesoderm lineage (S3 Fig). While the three T-box family members identified for MPS have a varying degree of literature support, all are driven by functionally coherent ontology terms, such as abnormal germ layer morphology (MP:0014138) and abnormal mesoderm morphology (MP:0014141) (S5b Table), offering attractive hypotheses.

In paraxial mesoderm, we found WNT signaling TFs, which promote paraxial and suppress lateral mesoderm cell fate (Fig 3c) [40]. We also find HOXC13, which is known for its necessary role in the proper development of the paraxial mesoderm into the presomatic mesoderm [43]. In early somites, we found MEIS3 and ZIC2, which are required in neural crest invasion and development of somite cells, respectively (Fig 3d) [44,45].

In lateral mesoderm, we found multiple GATA family members, of which GATA4 is a downstream effector of BMP signaling in lateral mesoderm (Fig 3e) [46]. We also saw *RUNX3*, which is co-expressed with *RUNX1* in lateral mesoderm [47]; both are necessary for hematopoiesis [37,39]. GLI1, a key TF in the hedgehog (HH) signaling, is essential for establishing left-right asymmetry in lateral mesoderm [48]. In cardiac mesoderm, we found FOS TFs, GATA TFs, and GLI1 (Fig 3f). Interestingly, FOSL2 regulates the rate of myocardial differentiation [49], and HH signaling via GLI1 is required for secondary heart field development [50]. As mentioned above, GATA factors are canonical drivers of cardiac development. All the GATA family members identified for mesoderm development (GATA1, 2, 4, and 6) are implicated in Human cardiovascular diseases [2,27]. In MEME-ChIP results, in contrast, we found very different and much more general factors among the top hits, including CTCF, MYC, and the members of E2F and TFAP families. The notable exception was ZIC2, which was the only TF identified by both methods (Fig 3d). Taken together, when compared with MEME-ChIP [10], WhichTF yielded many more cell-type-specific TFs with supported functional relevance (Fig 3).

### WhichTF identifies potentially disease-relevant TFs

Transcriptional mis-regulation has a broad impact on human diseases [2]. To assess whether WhichTF can shed light on the transcriptional regulatory molecular basis of human disorders, we examined systemic lupus erythematosus (SLE) as a case study. SLE is a heterogeneous and chronic autoimmune disorder most prevalent in adult women between puberty and menopause with varying degrees of population prevalence across different ancestry groups, including elevated incidence and prevalence in non-European populations [51,52], with more than 40% estimated heritability [53,54]. Abundant autoantibody production is observed in SLE patients. B-cells, which produce antibodies, are one of the key cell types involved in the disease. Perturbation experiments on patient-derived B-cells are currently lacking but alternative approaches, including genetic studies, have implicated many TFs. Genome-wide association studies (GWAS) revealed the polygenic architecture of the disease with more than 130 identified associated loci, many of which are located in non-coding regions and implicate immune-related pathways, including production of self-antigens, activation of the innate immune system, and dysfunction of the adaptive immune system [52,55,56]. Genetic and epidemiological studies suggest gene-environmental interactions play a role in SLE [52]. A recent study, based on a systematic co-localization assessment of GWAS-associated loci and experimentally measured TF occupancy, highlights a disease mechanism in which DNA-binding of Epstein-Barr virus EBNA2 protein and host-encoded co-factors results in transcriptional alteration [57]. While many TFs are known to play a role in the disease [52,57–61], most studies were performed on cell lines or mouse models, resulting in a limited understanding of the transcriptional dysregulation in clinical samples.

To better understand the regulatory landscape of SLE in patient-derived samples, we focused on ATAC-seq datasets from fresh and biobanked specimens of SLE patients and healthy controls [62]. Specifically, we sought differentially dominant TFs in healthy B-cells (healthy controls, HC) compared to SLE-affected B-cells and vice versa by applying both MEME-ChIP [10] and WhichTF. From MEME-ChIP differential analysis, we found that Sp factors of the Sp/XKLF TF family are ranked as the top hits for both directions of differential analyses comparing SLE and HC cells with very strong statistical significance (E-value < 1 x 10^−1300^ in each) (Table 3). SP1 and other Sp subfamily members are well-known versatile TFs that pervasively bind to GC-rich promoter regions [63].

**Table 3:** WhichTF identifies differentially dominant TFs from ATAC-seq measurement of B-cells from systemic lupus erythematosus (SLE) patients versus healthy controls (HC).

**a.** Healthy control vs. Systemic lupus erythematosus samples						
	WhichTF				MEME-ChIP	
	TF	-log_10_(CP)	Importance	PMID	TF	E-value
1	BCL6	28.7	Confirmed	34521812	SP2/SP1	9.5 x 10^−2800^
2	TFAP2B	19.3	Novel		ZBTB7B/SP4/ZN770	1.0 x 10^−841^
3	ZEB1	16.6	Suggestive	20856809		
4	ZSCAN21	14.7	Novel			
5	ZSCAN20	14.2	Novel			
**b.** Systemic lupus erythematosus vs. Healthy control samples						
	WhichTF				MEME-ChIP	
	TF	-log_10_(CP)	Importance	PMID	TF	E-value
1	GLI1	19.7	Suggestive	26552406	SP4/SP2	5.0 x 10^−1368^
2	ZNF143	11.0	Novel		HIC1/ZFX/ZFY	7.3 x 10^−682^
3	TCF7L2	6.0	Suggestive	31409726	NFATC3	5.5 x 10^−4^
4	ONECUT2	5.2	Suggestive	28317889	PAX3	2.8 x 10^−3^
5	DMRTC2	3.8	Novel		ZFP957	3.7 x 10^−3^

The top 5 TFs based on the analysis of (a) HC with respect to SLE (HC minus SLE) and (b) vice versa (SLE minus HC) are shown with the corresponding statistical significance, negative log_10_ conditional probabilities, -log_10_(CP). The results from a MEME-ChIP differential analysis are shown for comparison. The E-value is a statistical significance reported from MEME-ChIP and it represents the expected number of motifs in the randomized background set (Methods). The importance and PubMed ID (PMID) columns indicate whether the literature supports the identified TFs. WhichTF uniquely highlights TFs with known or suggestive functional relevance underlying transcriptional mis-regulation in complex autoimmunity traits.

With WhichTF, in contrast, we predict BCL6 as a differentially dominant TF in healthy vs. SLE B-cells (Table 3a). BCL6 is a well-documented transcriptional repressor required for mature B-cells to differentiate into antibody-producing plasma cells or memory B cells in the germinal center (GC) [64]. Given the roles of BCL6 along with BLIMP1 and AIM2 in B-cell differentiation [64–66], a recent study implicates this regulatory pathway as a novel target for SLE treatment [66]. In SLE B-cells versus healthy cells, WhichTF identified differentially dominant TFs implicated in autoimmune disorders (Table 3b). A sonic hedgehog (SHH)-Gli signaling pathway member GLI1 is involved in the pathogenesis of rheumatoid arthritis through synovial fibroblast proliferation [67]. A common intronic variant in *TCF7L2*, rs7903146, is a well-known causal risk allele for type 2 diabetes. A recent study suggests that this variant is also associated with latent autoimmune diabetes in adults [68]. In a model system to study multiple sclerosis, ZEB1 is suggested as a regulator of experimental autoimmune encephalomyelitis [69]. When we focus on the TFs implicated by previous studies–for example, IRF5, IRF7, IRF8, ETS1, NFKB1, STAT1, STAT4, YY1, IKZF1, IKZF2, SPI1 (a.k.a. PU.1), TBX21, BCL6, PRDM1 (a.k.a. BLIMP), PAX5 and AIM2 (of which, STAT4, PRDM1, PAX5, and AIM2 are not included in our PRISM library)–and investigated their rankings from WhichTF, we find BCL6 is the only TF ranked within top 5 in WhichTF. Though novel factors are potentially interesting, this also points to the need for larger sample size in epigenomic profiling of patient-derived cells (Discussion). None of the autoimmunity-associated TFs was identified using MEME-ChIP (Table 3), highlighting our ability to provide insights on the underlying transcriptional regulatory basis of the disease and candidate TFs worthy of further study.

### WhichTF uncovers stress response signatures

Context-specific measurements of open chromatin typically require purification of the desired cell type through mechanical and enzymatic tissue dissociation, which can be quite taxing on the cells. Indeed, it has been reported that stress response factors are often highly expressed in dissociated tissues [70]. Corroborating these observations, WhichTF often identifies canonical stress-associated TFs as some of the most dominant TFs in multiple very different contexts. As an illustration, we present WhichTF results for additional DNase-seq datasets (Table 4). For three endothelial cell types and adrenal gland cells, we found many FOS/AP-1 and NF-κB TFs, which are known for their roles in stress response. Even in the samples dominated by stress-associated TFs, we still found well-known context-specific players among the top hits, such as GATA3 and WT-1 in kidney cells and SOX and FOX TFs in endothelial cells [71–73]. We also found that the boundary between stress response and cell-type-specific functions can be ambiguous or context-dependent. For example, we found FOS/AP-1 and NF-κB dominant in keratinocytes and B-cells, respectively, which, in addition to being stress-associated, are also known for their respective context-specific functions [23,74].

**Table 4:** WhichTF identifies pervasive signatures of stress response TFs.

	B-cell			Keratinocyte			Adrenal Gland			Lymphatic Vessel Endothelium			Pulmonary Artery Endothelium			Dermis Vessel Endothelium		
	ENCFF719GOE			ENCFF047IIB			ENCFF212TPU			ENCFF354CZP			ENCFF596PRJ			ENCFF908DMH		
1	SPIB	f		FOSB	f	r	ZNF410			NFKB1		r	FOSL1		r	NFKB1		r
2	NFKB1	f	r	FOS	f	r	FOS		r	FOS		r	FOS		r	FOS		r
3	RELB	f	r	FOSL1	f	r	FOSL1		r	FOSL1		r	FOSL2		r	FOSL1		r
4	RELA	f	r	JUND	f	r	NFKB1		r	RELB		r	NFKB1		r	RELA		r
5	SPIC	f		BATF		r	JUNB		r	BATF		r	JUND		r	FOSL2		r
6	SPI1	f		FOSL2	f	r	FOSL2		r	JUND		r	RELB		r	BATF		r
7	ZNF410			BACH2		r	BACH1		r	FOSL2		r	BATF		r	FOSB		r
8	RUNX3			JUNB	f	r	JUND		r	REL		r	RELA		r	RELB		r
9	REL	f	r	BACH1		r	RELB		r	RELA		r	SOX10	f		JUND		r
10	STAT2	f		JUN	f	r	BACH2		r	SPIC	f		FOSB		r	SOX7	f	
11	WT1			NFE2L2			GATA3	f		FOSB		r	BACH2		r	ZNF410		
12	SNAI3			NFKB1		r	JUN		r	ZNF410			BACH1		r	BACH1		r
13	ZEB2	f		MZF1			WT1	f		SPIB	f		GATA4	f		GATA4	f	
14	ATF6			RELB		r	BATF		r	SOX30	f		JUNB	f		SOX12	f	
15	E2F5	f		ZFP217			NFE2L2			SOX7	f		GATA5	f		FOXD1	f	
16	IKZF3	f		ETS2	f		GATA6	f		SOX18	f		SOX30	f		FOXJ3	f	
17	ELF5			PITX1			FOSB		r	JUNB		r	SPIB	f		SOX30	f	
18	SP100			ATF6			GATA4	f		SOX12	f		SOX18	f		SOX18	f	
19	IRF9	f		TFCP2L1			MITF	f		BACH1		r	JUN	f		FOXO6	f	
20	SNAI1			MYC	f		FOXP2	f		FOXO3	f		FOXO3	f		FOXO4	f	

The top 20 TFs identified by WhichTF are shown in ranked order for six different cell types. TFs known to be involved in stress response are marked with ‘r’, while TFs in families known to be functionally important in each context are marked with ‘f’.

## Discussion

We present WhichTF, a novel ontology-guided computational method to identify and rank known or novel dominant TFs with functional relevance in any given set of accessible chromatin regions or through pairwise differential analysis of related samples. Our approach is broadly applicable across different species, cell types, and assays. The WhichTF score is calculated based on the functionally informed stratified enrichment of conserved TFBSs and built on high-confidence PRISM-predicted conserved TFBSs [9], gene regulatory domain annotations from GREAT [4], and curated biological knowledge in MGI phenotype ontology [26]. Applying WhichTF to dozens of samples across diverse biological contexts, we find a reassuring mixture of literature supported cell-type-specific TF functions, along with exciting suggested or novel predictions. Our differential analysis can help understand how closely related cell types diverge. We also show that our predictions are stable over a wide set of parameter choices.

To build WhichTF, we perform updates of GREAT [4] and computational prediction of conserved TFBSs with PRISM [9]. GREAT predicts candidate functional roles for sets of genomic regions by computing the statistical enrichment of ontology annotations within these regions without relying on TFBSs [4]. PRISM, on the other hand, uses sequence motifs of TFs to predict genome-wide TFBSs while accounting for local conservation from multiple alignment and does not rely on ontology annotations [9]. The TFBS prediction in PRISM is applied for each TF independently and is not designed to assess the cell-type-specific roles of TFs, nor suitable for ranking. In WhichTF, we specifically designed a TF hypergeometric test, a new statistical test to rank TFs (Methods). We combined the results from the TF hypergeometric test and the binomial enrichments as in GREAT to derive the WhichTF score. Our results highlight the values in the integration of two complementary approaches.

In contrast to many previously published computational methods that evaluate the statistical significance of the sheer abundance of molecular events without modeling their functional consequences, WhichTF integrates curated knowledge through ontology terms to link the accessible and conserved TFBSs to their potential influence on phenotypic abnormalities and to use such functional information to rank TFs. The benefit of leveraging ontology databases also comes with a potential cost. Given the biased coverage of ontology annotations [75], there is no guarantee that the biological contexts of interests are well represented in the ontology terms. When investigating the samples with ample ontology annotations, our closed-loop analysis [9] (S1 Table) may illuminate key ontology terms relevant for ranking TFs. Quantification of the degree to which the bias in the ontology annotations results in biased TF ranking is an important area of future investigation and an important consideration for one using WhichTF in their own domain. Empirically, our results indicate that the use of functional information in the MGI phenotype ontology [26] enabled us to focus on TFs whose perturbations cause clear phenotypic effects. Through comparison with existing methods, we find clear advantages of our approach in identifying TFs with cell-type-specific functions over standard methods, which tend to discover TFs with broadly shared roles across many cell types. Some existing tools, such as MEME-ChIP [10], RGT [11], Homer [12], cisTarget [13], and LOLA [14], implement motif enrichment analysis by assessing the enrichment of TFBSs in the user-provided accessible regions relative to the genome-wide background sets, while other approaches, such as the ones implemented in cisTarget [13], ChIP-Atlas [15], BART [16], and LOLA [14], evaluate the overlap between the user-provided accessible regions and previously characterized ChIP-seq datasets. Other methods like Lisa and Beta from Cistrome [17,18] ask related questions by focusing on the driver TFs within the observed molecular data. None of those existing tools use the curated functional knowledge to identify TFs. On the other hand, curated knowledge in ontologies is routinely used in gene-set enrichment analysis [76–78], but such analysis is not designed for functional analysis of non-coding genomes. A functionally guided approach like ours expands the application of curated knowledge in the functional analysis of non-coding regions of the genomes. It enhances the interpretation of the results because the functional information naturally provides a way to inspect ontology terms that drive the ranking of TFs, which is especially relevant to exploring under-characterized samples.

Many TFs regulate cell-type-specific functions in some cellular contexts, which may not be fully captured by a single method alone. One experimental method, ATI assay [31], is shown to offer complimentary cell-type-specific TFs or TF families to ours, though it requires additional bench work. Systematic identification of the complete set and composition of TFs that regulate cellular fates and cellular functions across diverse cellular contexts is still an open question. A future extension of the work that explicitly models TF dimers and co-factors, with the potential help of structural information [79], may provide additional insights into the composition of the TFs acting in given cellular contexts. We envision that computational and experimental approaches will play complementary roles.

In our differential analysis, we investigated the TFs implicated by differentially accessible peaks in multiple examples: comparing B-cell and T-cells (Table 2), mesoderm development (Fig 3), and SLE application (Table 3). In our T-cells vs. B-cells comparison analysis, for example, we saw the NF-κB TFs, including NFKB1, REL, and RELB, among the top-ranked differential TFs. Those TFs are known to be important for both T-cells and B-cells [23]. While those TFs are part of the same pathway, the differentially accessible regions driving the TF ranking are cell-type-specific. Indeed, literature supports both common and B and T-cell specific roles for NF-κB TFs [23].

In our application of WhichTF to patient-derived samples of SLE, we observed that the TFs identified from our approach had limited overlap with the TFs implicated by GWAS studies. Compared to the GWAS analysis typically conducted with thousands of case individuals, the ATAC-seq dataset used in the SLE analysis consists of four patients and four control samples. To reduce the potential biases from biobanking of the samples, we used only one patient sample in SLE vs. health control analysis. Given that SLE is a multifactorial trait and is affected by gene-environment interaction [52,57], a much larger sample size with a detailed phenotypic description would be beneficial to characterizing the regulatory landscape of patient-derived cells and to inferring the regulatory factors influencing disease-associated transcriptional states. As future studies make open chromatin datasets increasingly available (for example in [80]), we envision our approach, directly operating on specific cellular states in patient-derived samples, will play a complementary role to genetic studies that typically consider aggregated effects across cell types for the analysis of complex traits.

Among the top-ranked TFs in multiple human cell types, we find an under-characterized Zinc finger protein, ZNF410 (also known as ZFP410) (Table 4). It has been recently reported that in human erythroid cells, ZNF410 directly activates *CHD4* by binding to two clusters of conserved TFBSs in its promoter regions to facilitate the transition from fetal to adult hemoglobin. Of note, the authors reported [81] only eight genome-wide ChIP-seq peaks for ZNF410, all of which are located within the promoter region of *CHD4*. Interestingly, PRISM [9] predicts 2,894 conserved TFBSs for ZNF410 genome-wide, but GREAT [4] enrichment analysis using our flat ontology of Ensembl genes [82] does predict *CHD4* to have the strongest concentration of predicted binding sites (23 out of 2,894, GREAT binomial p-value of 1.4x10^-52^). However, *CHD4* continues to be our top predicted gene even when restricted, for example, to the cell-type-specific accessible regions in the adrenal gland (22 out of 61 accessible conserved ZNF410 TFBSs, GREAT binomial p = 5.7x10^-87^). Our predictions complement the experimental presence of thousands of ChIP-seq peaks for ZNF410 in an erythroleukemia cell line [83] and a broad ZNF410 expression in both human and mouse cell types, suggesting ZNF410 likely serves additional roles in *CHD4* regulation and broader cellular contexts.

In some cases, WhichTF identifies the specific TF within a family. In contrast, in other cases, identifying exact TFs within a TF family can be challenging, given the intrinsic similarity of DNA binding motifs within a TF family and the similar motif-based prediction of TFBSs for the family members. For example, WhichTF ranked GATA 4, 5, and 6 higher than GATA 1, 2, and 3 for the heart, while all the six GATA family members that share similar motifs are ranked within the top ten in erythrocyte progenitor cells (S6 Table), where GATA1 and GATA2 have known roles in erythrocyte development [84]. Further integration of additional modalities, such as RNA and protein expression profiles, collected from the same biological context (i.e., matched cell type and cellular state), may aid the identification of the TF family member with similar motifs.

While we have focused on chromatin accessibility data, this is by no means the only modality by which one can probe TFs acting in given cellular contexts. While our single input set requirement has its advantage in broader applicability, further integration with additional modalities, such as RNA-seq and ChIP-seq datasets collected from the same biological context may further enhance our ability to identify functionally important TFs. When the number of reads supporting the accessible regions is available in the differential analysis, an approach that considers the statistical significance of the differential accessibility like the one implemented in DiffBind [85] will enhance the power to detect the differentially dominant TFs. The modular design of WhichTF offers flexibility in incorporating the advancements in reference data. While the closest-gene works as a reasonable approach to assign putative downstream genes [86,87], cell-type-specific enhancer-gene links–from chromatin loops [86] or epigenomic-transcriptional correlation [88]–may complement cell-type agnostic proximity-based regulatory domain models in GREAT [4]. Our motif library used in the TFBS predictions with PRISM [9] covers all 12 major TF families where the number of TFs per family ≥ 1% of the total number of TFs (S1 Fig). Still, TFs not covered in the current motif library may have pivotal roles. Future expansion of the TF motif library and TFBS predictions as well as complementing cell-type agnostic TFBS predictions with experimentally measured TF binding profiles from ChIP-seq may enhance WhichTF’s predictive power. Furthermore, when combined with single-cell accessibility profiling [8] and large-scale projects, such as the Human Cell Atlas [89], WhichTF may aid the systematic characterization of dominant TFs across a spectrum of cell types. While WhichTF is not designed for sparse accessibility measurements from single-cell ATAC-seq profiling at the single-cell resolution, future extension of the approach to in silico cell-sorted pseudo-bulk accessibility profiles may reveal TFs with cell-type-specific functions from the same tissue.

The resources made available with this study [90], including WhichTF and the GREAT version 4 update, provide an excellent foundation for investigating the molecular mechanisms of TF-mediated cis-regulation. Together, these results highlight the benefit of combining experimental characterization of chromatin accessibility, high-quality TFBS reference datasets, and the genomic region annotation from curated functional information in biomedical ontologies and suggest that systematic identification of dominant TFs across many samples can be a powerful approach to understand molecular mechanisms of gene regulation and their influence on cell type differentiation, development, and disease.

## Methods

### GREAT v.4.0.4 update

We performed a major update of the popular Genomic Regions Enrichment of Annotations Tool (GREAT) [4] and released it as version 4.0.4. GREAT v4 supports the human (*Homo sapiens* GRCh38 and GRCh37/hg19) and mouse (*Mus musculus* GRCm38/mm10 and NCBIM37/mm9) genomes, using the following Ensembl [82] gene sets:

- Human GRCh38: Ensembl version 90
- Human GRCh37: Ensembl for GRCh37 version 90
- Mouse GRCm38: Ensembl version 90
- Mouse NCBIM37: Ensembl version 67

By focusing on genes with at least one Gene Ontology [91,92] (GO) annotation as described before [4], we defined putative gene regulatory domains for 18,777 (GRCh38), 18,549 (GRCh37/hg19), 21,395 (GRCm38/mm10), and 19,996 (NCBIM37/mm9) genes’ canonical transcription start sites.

We also updated the ontology reference data. GREAT currently supports the most recent versions of the following ontologies at the time of analysis: Ensembl genes [82], Gene Ontology (GO) [91,92], human phenotype ontology [93], and mouse genome informatics (MGI) Mammalian phenotype ontology [26] (S7 Table). The new Ensembl genes ontology is a “flat” ontology that makes every gene into a term, facilitating the testing of cis-regulatory elements congregation in the regulatory domains of individual genes. For MGI phenotype ontology, we mapped MGI gene identifiers to Ensembl human gene IDs using one-to-one ortholog mappings from Ensembl Biomart [82] version 90. In total, we compiled 2,861,656, 2,846,384, 2,734,172, and 2,675,691 gene-term relationships for GRCh38, GRCh37, GRCm38, and NCBIM37 genome assemblies, respectively (S7 Table).

### Computational TFBS prediction with PRISM

We also updated our computationally predicted PRISM [9] conserved transcription factor binding sites (TFBSs) for the human (*Homo sapiens* GRCh38 and GRCh37) and mouse (*Mus musculus* GRCm38 and NCBIM37) genomes. We took a manually curated TF monomer motif library consisting of 672 motifs from 567 TFs as in [94,95] (S1 Data). We then applied PRISM to predict TFBSs based on the evolutionary conservation of TF motif matches [9]. The GRCh37 and NCBIM37 tracks were each derived from GRCh38 and GRCm38 tracks, respectively, using liftOver [96].

We used the following multiple alignments from the UCSC genome browser [96]:

- Human GRCh38: hg38 100-way conservation alignment (lastz)
- Mouse GRCm38: mm10 60-way conservation alignment (lastz)

We removed Killer whale (*Orcinus orca*, orcOrc1) from the human alignment because of a chromosome name mismatch. We further subset the alignments to Eutherian species [9], resulting in 57 and 40 species for human and mouse, respectively. Using our manually curated TF monomer motif library [79], we applied PRISM [9] with default parameters and focused on the top 5,000 predicted TFBSs for each TF in our analyses. We used GNU Parallel in our analysis [97].

Using a TF family classification based on the DNA binding domain [1], we checked the coverage of our TF motif library. In a recent review paper [1], it has been estimated that there are 1,639 TFs in human. Our library covers all 12 major TF families where the number of TFs per family ≥ 1% of the total number of TFs (S1 Fig).

### PRISM TFBS enrichment method without functional annotation

We computed the binomial p-value of each TFBS set, using the total number of TFBS predictions, the number intersecting the query, and the fraction of the genome covered by the open chromatin region. We ranked the TFs by their binomial fold and labeled the results as PRISM enrichment in (Table 1).

### WhichTF analysis protocol

WhichTF combines user-specified accessibility measures, such as ATAC-seq or DNase-seq peaks, with pre-computed reference datasets to produce a ranked list of context-specific, dominant TFs. The reference datasets consist of GREAT regulatory domain models, MGI mammalian phenotype ontology-based gene annotations, and PRISM TFBS predictions.

WhichTF first identifies the top 100 ontology terms, (*π*_1_, …,*π*_100_), based on the GREAT enrichment tests [4]. Specifically, WhichTF takes the genomic elements in the input query set that overlap with the PRISM-predicted TFBSs and applies GREAT with the default “basal plus extension” association rule and a filter that terms must be associated with no fewer than two and no more than 500 genes. GREAT enrichment consists of two statistical tests, GREAT binomial and GREAT hypergeometric tests, and enables evaluating the enrichment of an input query region while accounting for a variable length of regulatory domains and ensuring that the reported regulatory enrichment is observed across the regulatory domains of multiple genes [4]. For each TF in the PRISM TFBS prediction library of *N* TFs, WhichTF takes an intersection of the TFBS prediction track and the user-submitted open regions using overlapSelect [96].

Each TF in the PRISM library has a different number of TFBSs and regulatory domains of different total sizes associated with each term. To capture the relative importance of different TFs within different contexts, WhichTF computes two measures of statistical significance for each TF and term and summarizes these measures in TF by term summary statistic matrices. Specifically, we apply the hypergeometric and binomial tests defined below:

#### TF hypergeometric test

Let’s define the GREAT gene regulatory domain for a term *π*_*j*_ as RegDom_j_, PRISM TFBS prediction for a TF_*i*_ as TFBS_*i*_, and user’s input query as QUERY. We define *n*_*i*_, *k*_*ij*_, *N*, and *K*_*ij*_ as follows:

$$n_{i}=\#\left\{{\text{TFBS}}_{i}\cap \text{QUERY}\right\}$$

$$k_{ij}=\#\left\{({\text{TFBS}}_{i}\cap \text{QUERY})\cap {\text{RegDom}}_{j}\right\}$$

$$N=\#\left\{\left({⋃}_{k}{\text{TFBS}}_{k}\right)\cap \text{QUERY}\right\}$$

$$K_{j}=\#\left\{(\left({⋃}_{k}{\text{TFBS}}_{k}\right)\cap \text{QUERY})\cap {\text{RegDom}}_{j}\right\}$$

Where ∩ denotes genomic intersection operation and #{*G*} denotes the number of elements in a genomic region set, *G*. With these parameters, we compute the hypergeometric p-value for each pair of TF_*i*_ and term *π*_j_:

∑k=kijmin(ni,Kj)KjkN-Kjni-kNni

#### TF binomial test

Using the intersection track, TFBS_*i*_ ∩ QUERY, we compute the GREAT binomial p-value for each pair of TF_*i*_ and term *π*_*j*_:

∑k=kijninikpπjk(1-pπj)ni-k

Where *p*_*π*_ denotes the probability of drawing a base annotated with the term *π* from non-gap genomic sequences under the uniform distribution [4].

#### Adaptive TF significance threshold

To eliminate false positives, WhichTF focuses on terms where the most significant TF is characterized by both a hypergeometric and a binomial p-value match. Using the enrichment statistics, WhichTF selects dominant TFs for each selected ontology term. We compute the adaptive threshold for each of the hypergeometric and binomial tests by finding a leap in the p-values of the top 10 TFs for each term using the following procedure. Let’s denote the top 10 hypergeometric p-values for a fixed functional term *π* as *p*_1_ ≤ *p*_2_ ≤ ⋯ ≤ *p*_10_. We define the difference of adjacent negative log of p-values as $d_{k}=-\text{log}\;\frac{p_{k}}{p_{k+1}}$. We define *m*, the index with the largest leap in p-value as *m* = argmax_*k*_ *d*_*k*_. Our adaptive threshold is *p*_*m*_ and we only keep TFs with hypergeometric p-values that satisfies *p* ≤ *p*_*m*_. We define the adaptive threshold for binomial p-values in the same way. We say TF_*i*_ is significant for the term π_*j*_ when it passes the adaptive thresholds for both TF hypergeometric and TF binomial tests.

#### WhichTF score computation via the sum of WhichTF partial scores

For each TF, WhichTF computes the score by the following equation. Let (*π*_1_, …,*π*_*K*_) be the set of terms selected from step 1 in the order of relevance with π_*1*_ as the top hit. Let Rank(TF_*i*_, π_*j*_) be the rank of the TF_*i*_ for term π_*j*_. Let Significant(TF_*i*_, π_*j*_) denote a Boolean variable that indicates whether TF_*i*_ passes the filters described above for the term *π*_*j*_ (i.e., “Significant” is one if the TF passes the significance filter and zero otherwise). With this notation, we define the WhichTF score of TF_*i*_ as the sum of partial scores across ontology terms:

$$\text{WhichTF}\;\text{partial}\;\text{score}({\text{TF}}_{i},\pi_{j})=\frac{\text{Significant}({\text{TF}}_{i},\pi_{j})}{\sqrt{j\cdot \text{Rank}({\text{TF}}_{i},\pi_{j})}}$$

$$\text{WhichTF}\;\text{score}({\text{TF}}_{i})=\sum_{j}\text{WhichTF}\;\text{partial}\;\text{score}({\text{TF}}_{i},\pi_{j})$$

The WhichTF partial score is a stratified enrichment metric, which quantifies the contribution of each ontology term to the final TF ranking. Given that the WhichTF partial score and WhichTF score are both nonparametric and both depend on function (approximated through the top 100 enriched ontology terms) and the conserved TFBS enrichment within the regulatory regions associated with those terms, the TF rankings are more interpretable than the individual TF scores.

#### WhichTF conditional p-values

WhichTF computes the statistical significance of a WhichTF score based on a null model that any ordering of TFs within each term is equally likely. Thus, the probability of a given score is determined by the relative number of configurations with the score. To enumerate the number of configurations with a given score in polynomial time, we devised a dynamic programming approach [98] which acts recursively on the number of functional terms, *K*. This procedure first discretizes each contribution to the summand in the definition of the WhichTF score defined above. Let $\left\{s_{j1},s_{j2},\ldots ,s_{jM_{j}}\right\}$ be the set of all the possible cumulative scores up to term *π*_*j*_, that is the scores gotten by computing the above sum only up to term *π*_*j*_. Here, *M*_*j*_ is the number of distinct discretized scores up to term *π*_*j*_. Let *n*_*ji*_ represent the number of different ways of getting each such score, *s*_*ji*_, and let $S_{j}=\left\{\left(s_{j1},n_{j1}\right),\left(s_{j2},n_{j2}\right),\ldots ,(s_{jM_{j}},n_{jM_{j}})\right\}$ be the set of all tuples of scores and number of configurations. Finally, let $\left\{t_{j1},t_{j2},\ldots ,t_{jM_{j}}\right\}$ denote the individual summands at term *π*_*j*_.

The p-value of each score is computed directly from *S*_*K*_, the full set of cumulative scores and number of configurations, by dividing the number of configurations with scores greater than or equal to a given score by the total number of configurations. This list of tuples, *S*_*j*_, can be computed recursively with the base case of S_0_ = {(0,1)}. The set of scores at level *j+1* is given by all combinations, *s*_*ji*_ + *t*_*j*+1*k*_, with the number of configurations given by aggregating over all combinations of *s* and *t* that yield the same cumulative score.

Given that the WhichTF scores of multiple TFs are not independent, we apply the procedure defined above from the top-scoring TF to the TF with the lowest score and compute conditional statistical significance. This means that for the computation of statistical significance of the *i*-th ranking TF, we remove TFs whose rank is smaller than *i* and apply the recursive procedure defined above. Of note, the conditional p-value of the top-ranked TF is exactly the same as the marginal (unconditional) p-value of the top-ranked TF.

### Robustness analysis

To investigate the effects of the number of input regions, we subsampled the genomic elements ranked by the SCORE column in the input BED file. We picked random subsets to break ties.

To investigate the effects of the length of the input regions, we set a series of maximum lengths of the input regions and trimmed each of the elements in the input BED files while preserving the midpoint of each element.

### Application of WhichTF in diverse functional contexts

We applied WhichTF in diverse cell types. For the top-ranked TFs, we manually performed literature review and evaluated whether the functional role of the identified TFs was reported in the literature. Specifically, we classified the confidence of the results into the following three categories:

1. Confirmed: existing literature supports the role of our predicted TF’s role;
2. Suggested: literature reports closely related factors, such as co-factors or functionally related family members, or the identified TFs in related context; or
3. Novel: we could not currently find supporting literature

#### Multiple cell types from the ENCODE/Roadmap project

From the ENCODE/Roadmap data portal, we obtained “hotspot” files derived from DNase-seq experiments [99,100]. All coordinates are provided in GRCh37. We present an analysis spanning 95 samples from 12 cell types and tissues (S8a Table).

We systematically applied WhichTF to each sample and obtained the ranked list of TFs as well as a vector of WhichTF scores across all TFs in the library. We applied t-SNE, a non-linear dimension reduction method [33], implemented in the *DimensionReduce* function in Mathematica [101] (Fig 2).

We applied WhichTF to mouse ENCODE DNase-seq datasets from the same four cell types used for the human analysis (S3 and S8b Tables). All mouse datasets used the GRCm38 reference assembly.

#### Cell type-specific expression analysis

We presented cell-type-specific RNA-seq data from the GEO database [38] (GSE118165). We focused on the unstimulated samples and plotted the expression of *SPIB* and *RUNX3* for lymphoid cells in T and B cell lineages.

#### WhichTF differential analysis

To find TFs dominant in an input set A compared to another input set B, we defined set A and set B regions as foreground and background, respectively. We used bedtools [102] “subtract” to keep a subset of A that does not overlap with B. We applied WhichTF single run mode (above) on the identified differentially accessible regions.

#### Mesoderm lineage dataset

Using ATAC-seq datasets SRP073808 from NCBI GEO database of mesoderm development [40] (S8c Table), we applied WhichTF differential analysis following the diagram of sequential differentiation.

#### Systemic lupus erythematosus (SLE) dataset

The ATAC-seq datasets described in Scharer *et al*. [62] were derived from freshly isolated or biobanked specimens of peripheral blood mononuclear cells. Eight sets (4 SLE and 4 healthy controls [HC]) were taken from the NCBI sequence read archive (SRA, S8d Table). No additional information on patient demographic and clinical information were available in this dataset. Paired-end reads were mapped using bowtie2 with the outer distance flag (-X) set to 1000 and otherwise default settings [103]. Samtools was used to generate a sorted bam file and MACS2 was used to call peaks with shift set to 37, extension size set to 72, and broad and keep-dup flags on [104,105]. Given that some of the samples in this dataset are from a biobank, we conservatively defined differentially accessible regions as shown below and applied WhichTF differential analysis (Table 3):

$$SLE–HC≔SRR3158183-{⋃}_{x\in SRR3158176-9}x$$

$$HC–SLE≔{⋂}_{x\in SRR3158176-9}x-{⋃}_{x\in SRR3158180-3}x$$

### Tissue-specific gene expression of the identified TF

Using the dataset phs000424.v7.p2 obtained from the GTEx Portal [106] on 05/24/2019, we investigated whether the identified TFs have a tissue-specific expression (S3 Fig).

### Comparison with existing TF ranking tools

We compared the results of WhichTF against MEME-ChIP from the MEME suite [10], a motif enrichment tool implemented in the regulatory genomics toolbox (RGT) [11], HOMER [12], cisTarget [13], and Genomic Locus Overlap Analysis (LOLA) [14] (Table 1 and S1 Table).

For the accessibility tracks from the four ENCODE human cell types in hg19, we applied MEME-ChIP [10], an integrated motif analysis pipeline, version 5.1.1. To maximize the coverage of motif libraries used in the enrichment analysis, we used 17 human and mouse motif libraries in their pre-computed motif database version 12.19 (S9 Table). We took the 200 bp regions surrounding the midpoint of each input region. We skipped SpaMo (Spaced Motif Analysis tool) with the ‘-spamo-skip’ option to keep the computation tractable and otherwise used default parameters. For differential analysis, we specified the negative set using the ‘-neg’ option. Given that the analysis pipeline includes motif clustering, we reported all identified TF names for up to 5 top enriched motifs. We included the E-value from the MEME suite, which is a measure of statistical significance, defined as the number of expected motifs with the matched size (motif width) and sites computed on a randomized background set.

Using the same set of accessibility tracks, we applied the motif enrichment pipeline implemented in the regulatory genomics toolbox [11]. This tool computes the fold enrichment and the corresponding p-values based on Fisher’s exact test via a two-step computational pipeline consisting of ‘rgt-motifanalysis matching’ and ‘rgt-motifanalysis enrichment.’ Given the large-memory footprint of the pipeline, we restricted our analysis to the top 5,000 regions in the input accessibility BED file and repeated the computational analysis ten times, using the score column with randomized tie-breaking to select the top 5,000 elements. We reported the top 5 identified TFs for each cell type based on the average rank of the ten runs.

We applied HOMER version 4.11 [12] for the same set of accessibility tracks using its reference data for hg19 assembly with the default parameters. We reported the top 5 unique TFs and regulators for “known motif enrichment” and “de novo motif discovery” followed by the motif similarity search with known PWMs.

To prepare the input regions set that meet the file size limit of cisTarget [13], we took the top 10,000 peaks of the same set of accessibility tracks ranked by the SCORE column in the input BED file. We applied cisTarget using their reference database version 6 with the default optional parameters. We reported the top 5 unique TFs and regulators, listed as “​​Possible TFs” in the output file, as well as the maximum value of their normalized enrichment score (NES) [13].

For Genomic Locus Overlap Analysis (LOLA) [14], we downloaded its Region Databases (LOLA core, version 20180412) and applied the enrichment analysis using the active DHS set as the background set using LOLA version 1.16.0. We applied the “redefineUserSets” function to the input query, followed by enrichment analysis with the “runLOLA” function. We reported the top 5 unique TFs and regulators.

We also applied MEME-ChIP [10] for B-cells vs. T-cells comparison, mesoderm development examples, and SLE dataset using the same parameters.

## Supporting information

S1 Fig Number of known and available TF genes in the WhichTF reference dataset. The number of known and available TF genes in the WhichTF reference dataset across major TF families based on Lambert et al, 2018 are shown across the 12 largest TF families.
(TIF)

S2 Fig Gene expression of the top identified TF genes from the differential WhichTF analysis applied on B and T-cell DNase-seq data. Gene expression of the top identified differential TF genes, SPI-B and RUNX3, are shown (horizontal axis) across diverse lymphoid cell types (vertical axis) for up to four healthy donors. SPIB has specific expression in B-cells, whereas RUNX3 has elevated expression in T-cells.
(TIF)

S3 Fig TBX15 shows the tissue-specific gene expression in muscle. The Human cell types are shown on the x-axis and the expression (Transcripts Per Million) is shown on the y-axis. The median, 25th, and 75th percentiles are shown as box plots. Individual data points are shown as outliers if they are above or below 1.5 times the interquartile range.
(TIF)

S1 Table WhichTF identifies cell-type-specific functionally important TFs in diverse cell types. This is an extended version of Table 1 with the additional methods included in the comparison. The top 5 identified TFs for B-, T-, heart, and brain cells are shown for eight methods: MEME-ChIP, regulatory genomics toolbox (RGT) motif enrichment tool, HOMER (enrichment for known motifs), HOMER (de novo motif discovery followed by similarity search to known motifs), LOLA, cisTarget, PRISM conserved binding site enrichment, and WhichTF. Here, -log10(P) denotes the statistical significance (negative log 10 p-value) of the TFBS enrichment; -log10(CP) denotes the statistical significance (conditional p-value, conditioned on the TFs ranked above, Methods); and PMID represents the PubMed ID.
(XLSX)

S2 Table WhichTF partial scores capture cell-type-specific TF functions. We show for each WhichTF top predicted TF in 4 different cellular contexts their highest supporting partial scores (Annotated column). Nearly 90% of these terms are annotated to the TF gene itself based on closed-loop validation (Methods). Here ENC indicates ENCODE accession id and MP is a prefix for MGI mammalian phenotype ontology.
(XLSX)

S3 Table WhichTF and active TF identification (ATI) results for mouse data. The ATI experiment identifies active TFs, by quantifying the total number of TF-DNA binding events in cells or tissues, performing sequencing and computational motif identification on TF-bound DNA to identify TF families, and distinguishing individual TFs through mass spectrometry. ATI identified active transcription factor families across five cell types (ES cells, heart, spleen, brain, and liver) and classified TFs into common TFs found in all five types, shared TFs found in more than two types, and specific TFs found in one or two cell types (PMID: 29786094). The top 5 WhichTF TFs with their corresponding ENCODE accession IDs and statistical significance (conditional probability), are shown for mouse (a) B-cells, (b) T-cells, (c) heart cells, and (d) hindbrain cells. ATI results are shown for the two most overlapping cell types, heart and brain. The importance and PubMed ID (PMID) columns indicate whether (i) existing literature supports the identified TFs (confirmed); (ii) literature reports closely related factors, such as co-factors and functionally related family members, or the identified TFs in a related context (suggestive); or (iii) novel. For the ATI assay, we report importance as identified in their original publication [PMID: 29786094]. WhichTF and ATI highlight mostly confirmed but largely complementary results.
(XLSX)

S4 Table WhichTF robustness analysis. (a) WhichTF ranking is robust to input region sub-sampling. The top 5 identified TFs are shown for human B-cells, T-cells, heart cells, and brain cells (sample name and the corresponding ENCODE accession ID are shown in the “Sample” column) across the different number of regions in the input files (70%, 80%, 90%, and 100%). The 100% corresponds to the original input file. For the other ones, we subsampled the elements in the BED file based on the SCORE column before applying WhichTF. (b) WhichTF ranking is robust to the lengths of the input region. The top 5 identified TFs are shown for human B-cells, T-cells, heart cells, and brain cells (sample name and the corresponding ENCODE accession ID are shown in the “Sample” column) across different maximum lengths of the regions in the input files (200 bp, 500 bp, 1000 bp, 2000 bp, and original). The ‘original’ correspond to the original input file and for the other ones, we trimmed, if needed, each element in the BED while preserving its midpoint before applying WhichTF. (c) WhichTF ranking is robust to the number of top enriched terms it uses. The top 5 identified TFs are shown for B-cells, T-cells, heart cells, and brain cells (sample name and the corresponding ENCODE accession ID are shown in the “Sample” column) across different numbers of top enriched ontology terms (50, 75, 90, 100 [default], 110, 125, and 150). The ‘100 [default]’ corresponds to the default parameter configuration of WhichTF (see Methods).
(XLSX)

S5 Table The top contributing ontology terms for the top five identified TF in mesoderm differential analysis. (a) Anterior primitive streak (APS) vs. Embryonic stem cells (hESCs). (b) Mid primitive streak (MPS) vs. Embryonic stem cells (hESCs).
(XLSX)

S6 Table The rank of GATA family members in heart and erythrocyte progenitor cells. The ranks of GATA family members in heart and erythrocyte progenitor cells from the human ENCODE dataset are shown.
(XLSX)

S7 Table The update summary of GREAT v4.0.4 ontologies. Ensembl genes is a flat ontology defined from the set of genes with at least one meaningful annotation in gene ontology (Methods).
(XLSX)

S8 Table Accession IDs used in the analysis. (a) Human ENCODE accession IDs (EID) used in our study and the corresponding cell type or tissue. (b) Mouse ENCODE accession IDs used in our study and the corresponding cell type or tissue. (c) Mesoderm development sample IDs, sample description, and the reference to the corresponding results. (d) Sequence read archive (SRA) accession IDs for the systemic lupus erythematosus (SLE) dataset. SLE indicates disease and HC indicates healthy control.
(XLSX)

S9 Table List of MEME motif datasets used in the comparison analysis. (XLSX)

S1 Data The list of TFs and motifs included in the PRISM library. The TF names, number of motifs, the list of motif names, and Ensembl gene IDs are shown.
(XLSX)

S2 Data The raw data used for Fig 2. (XLSX)
