# Supplementary figures and images for: WhichTF is functionally important in your open chromatin data?
Source: PLoS Comput Biol. 2022 Aug 30;18(8):e1010378. doi: 10.1371/journal.pcbi.1010378 (PMC9426921; doi:10.1371/journal.pcbi.1010378)

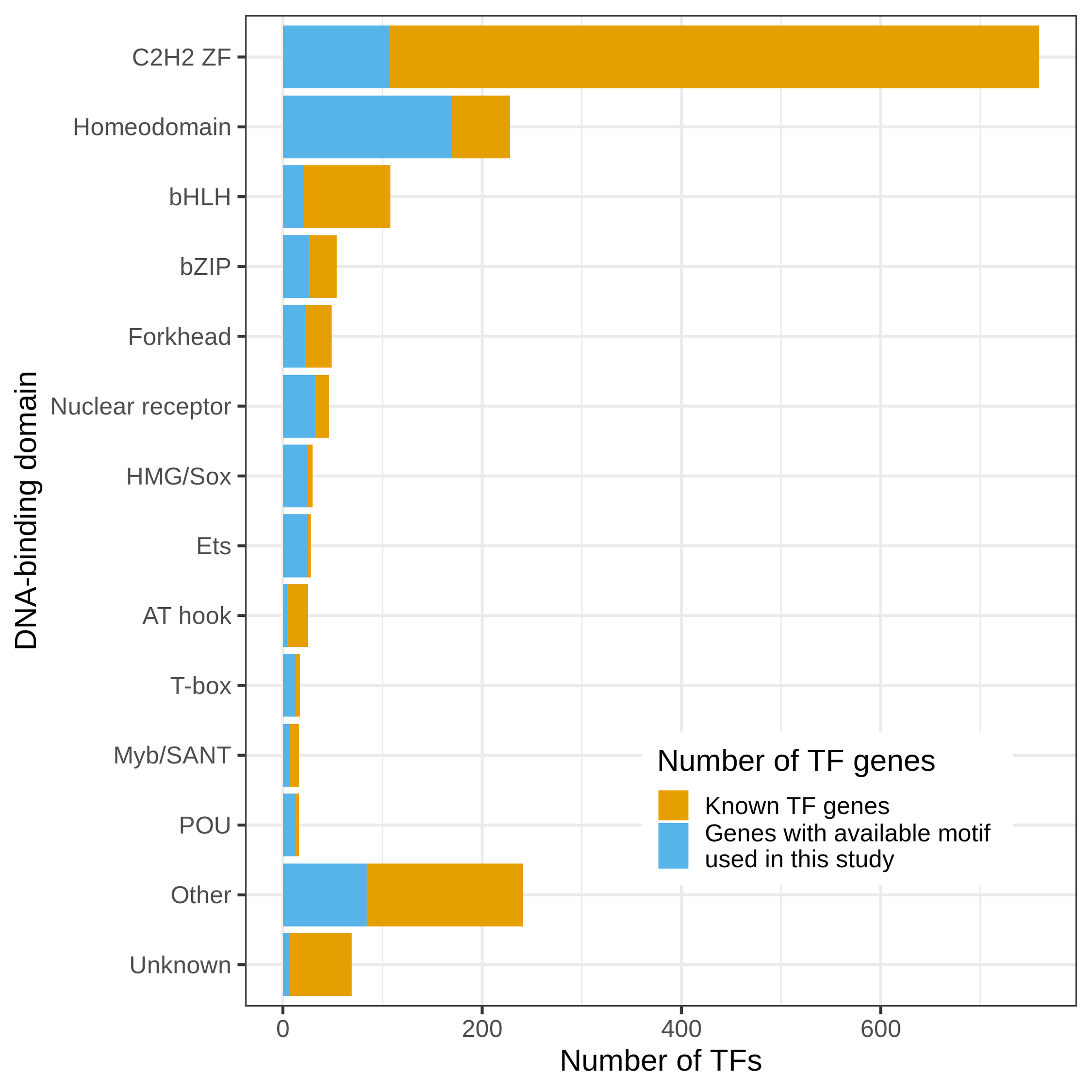

Supplement: S1 Fig — The number of known and available TF genes in the WhichTF reference dataset across major TF families based on Lambert et al, 2018 are shown across the 12 largest TF families. (TIF) [file pcbi.1010378.s001.tif]

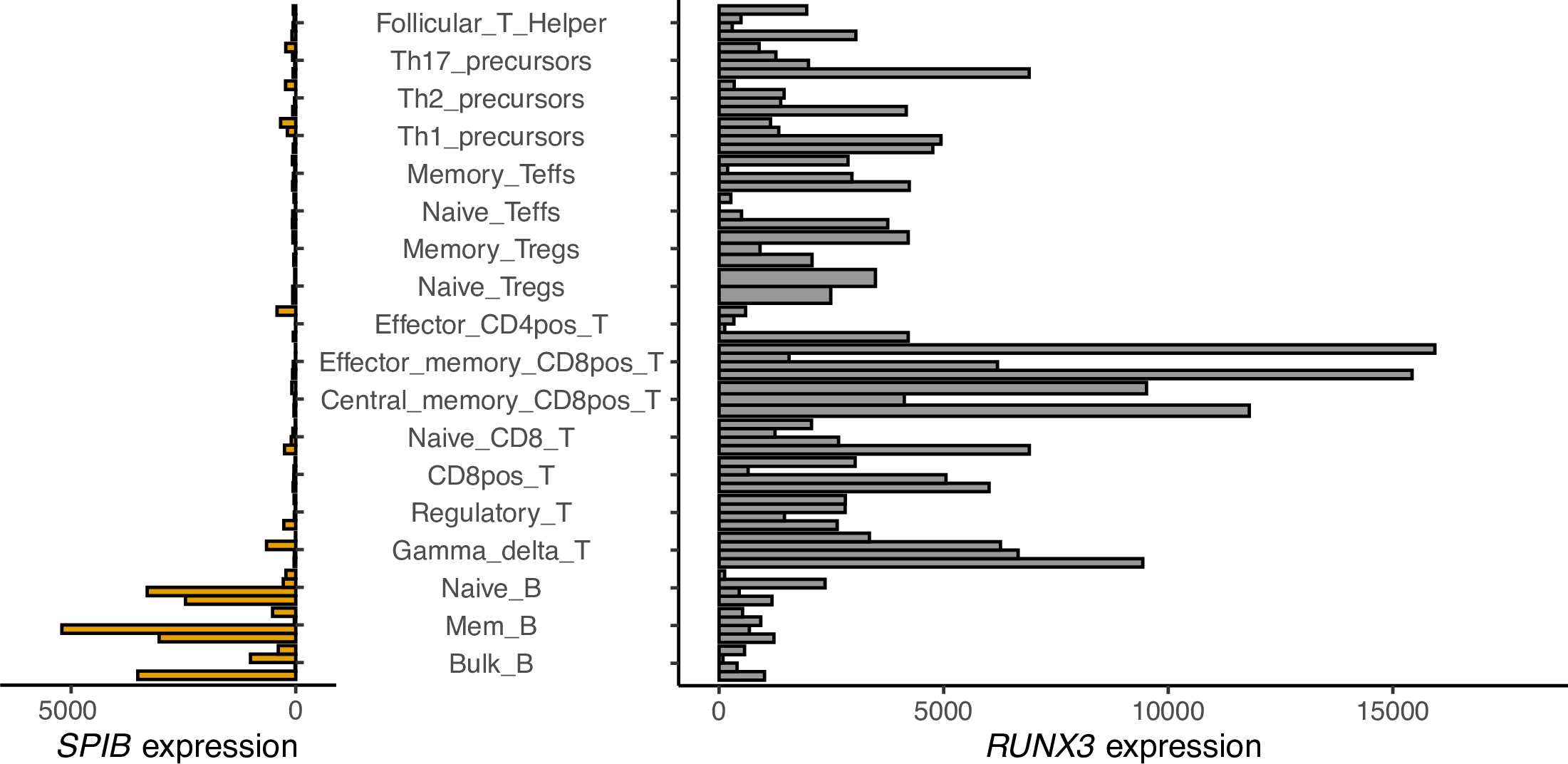

Supplement: S2 Fig — Gene expression of the top identified differential TF genes, SPI-B and RUNX3, are shown (horizontal axis) across diverse lymphoid cell types (vertical axis) for up to four healthy donors. SPIB has specific expression in B-cells, whereas RUNX3 has elevated expression in T-cells. (TIF) [file pcbi.1010378.s002.tif]

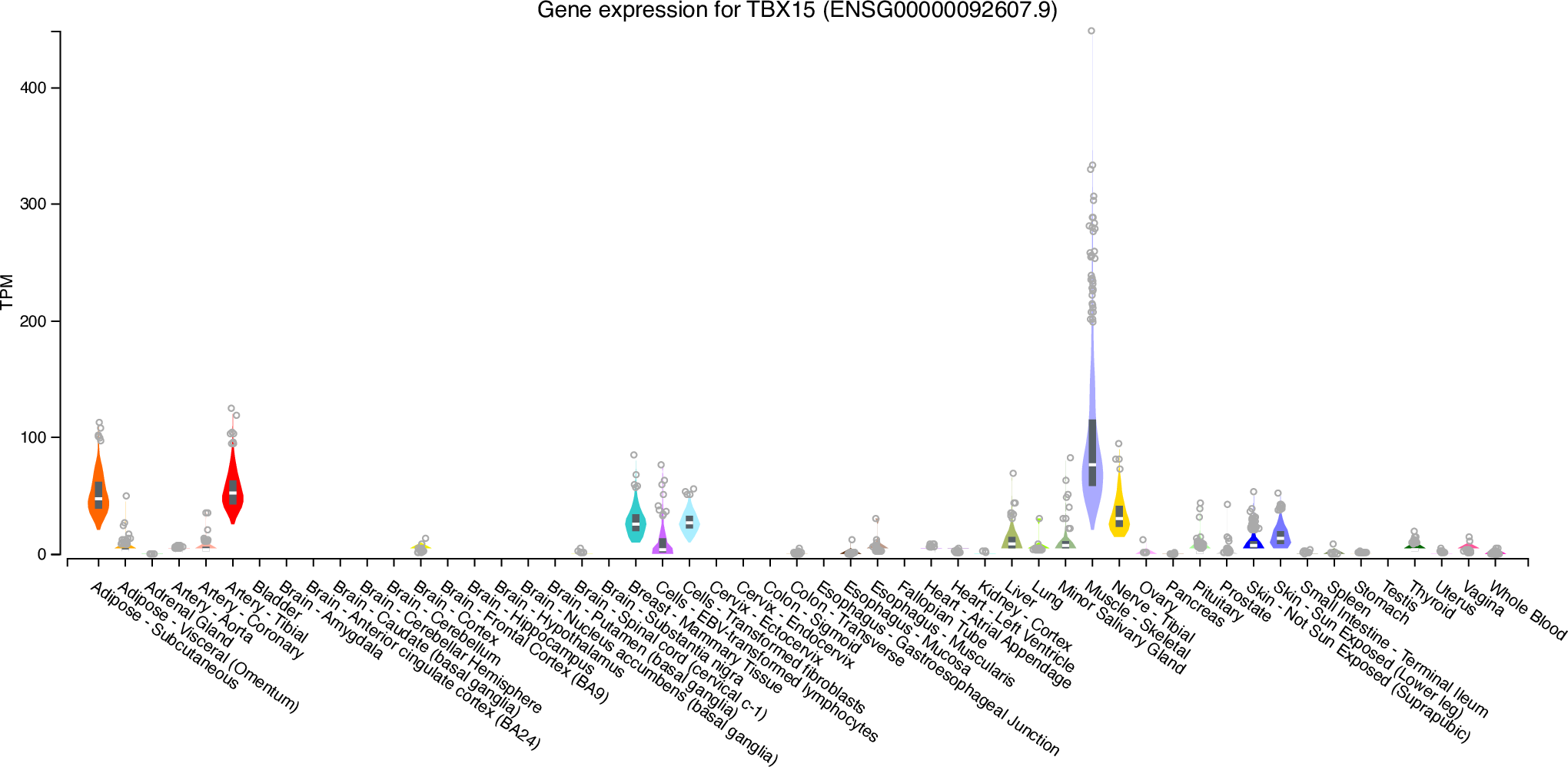

Supplement: S3 Fig — The Human cell types are shown on the x-axis and the expression (Transcripts Per Million) is shown on the y-axis. The median, 25th, and 75th percentiles are shown as box plots. Individual data points are shown as outliers if they are above or below 1.5 times the interquartile range. (TIF) [file pcbi.1010378.s003.tif]
